# Normozoospermic infertile men possess subpopulations of sperm varying in DNA accessibility, relating to differing reproductive outcomes

**DOI:** 10.1093/humrep/deaf081

**Published:** 2025-05-16

**Authors:** Mark E Gill, Manuel Fischer, Christian De Geyter, Antoine H F M Peters

**Affiliations:** Reproductive Medicine and Gynecological Endocrinology (RME), Universitätsspital Basel, University of Basel, Basel, Switzerland; Friedrich Miescher Institute for Biomedical Research (FMI), Basel, Switzerland; Reproductive Medicine and Gynecological Endocrinology (RME), Universitätsspital Basel, University of Basel, Basel, Switzerland; Reproductive Medicine and Gynecological Endocrinology (RME), Universitätsspital Basel, University of Basel, Basel, Switzerland; Department of Biomedicine (DBM), University of Basel, Basel, Switzerland; Friedrich Miescher Institute for Biomedical Research (FMI), Basel, Switzerland; Faculty of Science, University of Basel, Basel, Switzerland

**Keywords:** human reproduction, sperm, chromatin accessibility, pre-implantation embryo, ART, IVF, ICSI

## Abstract

**STUDY QUESTION:**

Can a reliable assay be developed to quantify DNA accessibility in human sperm to help with the assessment of pre-implantation development affected by dense packaging of mammalian sperm’s genetic material?

**SUMMARY ANSWER:**

We adapted NicE-view, an assay that directly labels accessible DNA, for use in human sperm and applied it to examine spermatozoa from infertile individuals with distinct reproductive outcomes.

**WHAT IS KNOWN ALREADY:**

Existing data suggest a connection between sperm chromatin compaction and reproductive outcomes. The assays used to generate this data, however, measure chromatin compaction indirectly and thus understanding their meaning is challenging.

**STUDY DESIGN, SIZE, DURATION:**

Between April 2020 to December 2023, 60 normozoospermic infertile men were invited to participate in an experimental study and asked to provide a semen sample.

**PARTICIPANTS/MATERIALS, SETTING, METHODS:**

Among the 60 individuals forty had undergone at least one treatment with ART. Among these ART-treated participants, 20 were included in the study because after fertilization only one or no embryos developed during embryo culture (low blastocyst growth rate, LBGR). The other 20 men were included as at least 50% of cultured embryos developed to the blastocyst stage (high blastocyst growth rate). Additionally, 20 previously infertile individuals obtained a pregnancy naturally (NATP) and were included as well. Washed spermatozoa obtained from seminal plasma or prepared by swim-up procedure were processed for NicE-view to determine DNA accessibility as a marker of chromatin condensation using confocal microscopy. Images of more than 3 million spermatozoa were acquired. Computer-assisted image segmentation was used to identify individual sperm heads and DNA accessibility levels were then quantified in each. We also compared NicE-view to chromomycin A_3_ (CMA3), a conventional marker of chromatin de-condensation, and ATAC-see, an alternative assay for measuring DNA accessibility.

**MAIN RESULTS AND THE ROLE OF CHANCE:**

Both semen and swim-up samples of participants contained two well-delineated subpopulations of spermatozoa with distinct DNA accessibility levels, the frequencies of which varied among individuals. Interestingly, individuals with high frequencies of highly accessible sperm DNA, as measured in semen, possessed decreased sperm concentrations. Moreover, participants with high frequency of highly accessible sperm DNA were more common in the LBGR sub-group. Surprisingly, selection of motile sperm by swim-up enriched for sperm with high DNA accessibility in participants from all three sub-groups. Chromatin accessibility measurements by Nice-view were distinct from DNA staining with the fluorescent CMA3 dye, and NicE-view allowed much clearer separation of sperm subpopulations than ATAC-see.

**LIMITATIONS, REASONS FOR CAUTION:**

This was a single-centre study with a cohort of 60 individuals. Sperm samples containing very high frequencies of sperm with increased DNA accessibility were more common in the LBGR sub-group. The number of individuals with this pattern was, however, limited, even within this category.

**WIDER IMPLICATIONS OF THE FINDINGS:**

High DNA accessibility is associated with poor pre-implantation embryonic development *in vitro* and NicE-view may be used for the prediction of abnormal embryonic development in ART. Further studies examining samples from larger cohorts of participants and the localization of accessible regions within the sperm genome are needed to fully evaluate the utility of this method.

**STUDY FUNDING/COMPETING INTEREST(S):**

Swiss National Science Foundation (Grant No. 189264), Swiss Center for Applied Human Toxicology (SCAHT) research (Grant No. 1 ‘male reproductive toxicity’) (both to C.D.G.), and the Novartis Research Foundation (to A.H.F.M.P.). M.E.G., C.D.G., and A.H.F.M.P. are authors on a patent application (EP23210754.0) on the use of NicE-view for the assessment of sperm.

**TRIAL REGISTRATION NUMBER:**

ClinicalTrials.gov ID NCT04256668.

## Introduction

Infertility is a widely prevalent condition and at least half of all cases are ascribed to male infertility (Thonneau *et al.*, 1991; Agarwal *et al.*, 2015). Reduced semen quality, as defined by either concentration, progressive motility, morphology of sperm, or of any combination, is the most used surrogate parameter that correlates with male infertility. The normal ranges of each parameter and any deviations were calculated by shorter or longer time to pregnancy in natural conception (Cooper *et al.*, 2010; World Health Organization, 2021). The current features of semen quality fail to explain all infertility, as was clearly demonstrated by several prospective follow-up studies of couples intending to achieve pregnancy (Bonde *et al.*, 1998; Zinaman *et al.*, 2000; Buck Louis *et al.*, 2014). In addition, in ART, most particularly following ICSI, failed treatment outcomes often cannot be explained adequately when relying on conventional semen analysis only (Sciorio and Fleming, 2024). Novel sperm function tests that can dissect the single steps of male contribution to reproduction are now needed to understand male infertility.

Sperm chromatin is highly compacted when compared to somatic cells, a feature thought to both protect the DNA from exogenous damage and to enable more efficient passage of the spermatozoa towards the oocyte. DNA fragmentation in sperm has been shown to decrease reproductive success both in natural conception and in ART. Differences in fertility applications, however, mean that this parameter is not standardized as a part of conventional semen analysis (Ribeiro *et al.*, 2017). Recent work in mice suggests that the type of DNA damage in sperm (single- vs double-stranded) influences the effects on embryonic development (Nguyen *et al.*, 2023). Both forms of damage in sperm decreased the development of embryos to the blastocyst stage, but single-stranded damage caused a more dramatic effect (Nguyen *et al.*, 2023).

In nearly all eukaryotic cells, the DNA is packaged using highly conserved histone proteins, while mammalian sperm is mostly packaged with small, basic DNA-binding proteins known as protamines (Pruslin and Rodman, 1985). Depletion of protamines from the nuclei of spermatozoa is associated with increased sperm DNA fragmentation and impairs fertility in both mice and humans (Cho *et al.*, 2003; Aoki *et al.*, 2005; Castillo *et al.*, 2011). Reduced packaging of sperm chromatin together with more DNA fragmentation has been linked to developmental arrest of pre-implantation embryos (Fatehi *et al.*, 2006; Burruel *et al.*, 2014; Simon *et al.*, 2014). An assay measuring abnormal chromatin packaging in human spermatozoa is needed to disentangle defects in oocytes from those in fertilizing sperm during early embryonic development.

Many studies focusing on sperm chromatin density have utilized indirect assays to identify changes in DNA packaging proteins. For instance, increased levels of chromomycin A_3_ (CMA3), a DNA-binding fluorescent dye, have been suggested to correlate with poor reproductive outcomes (Ni *et al.*, 2016). Staining by this dye in sperm has been associated with decreased protamination, though the evidence directly linking these features is limited (Bianchi *et al.*, 1993). Zygotes generated by ICSI from sperm enriched for strong CMA3 staining more frequently failed to decondense the sperm nucleus following fertilization (Sakkas *et al.*, 1996). Alternatively, acid aniline blue (AAB) has been used for many decades to measure ‘nuclear immaturity’ in sperm. AAB is a cytological dye that binds to the amino acid lysine, which is substantially more abundant in histones than protamines. Spermatozoa staining strongly with AAB have thus been thought to retain more histones in their chromatin than those staining weakly. However, confirmation that increased AAB staining is only associated with increased histone content is absent. Indeed, chromatin de-compaction associated with capacitation, which is not thought to increase histone content, also results in increased AAB staining, likely due to increased dye accessibility (de Lamirande *et al.*, 2012).

In addition to the challenge of direct molecular interpretation of these assays, the method of quantification of their levels is also potentially problematic. The standard to perform such assays involves examination of up to 200 sperm at the microscope, and assessment by eye of whether a sperm is positive or negative. Utilization of computational image analysis pipelines would allow dramatic increases in the number of sperm examined and objective quantification of exact signal levels in individual sperm.

Direct molecular assessment of chromatin density is now possible using a variety of approaches that enzymatically alter accessible DNA. One of these approaches includes the Assay for Transposase Accessible Chromatin (ATAC) methods: ATAC-seq (Buenrostro *et al.*, 2013) and ATAC-see (Chen *et al.*, 2016), where an engineered, hyperactive bacterial transposase (Tn5) integrates oligonucleotides into the DNA of accessible regions. ATAC-seq has been performed in human (Jung *et al.*, 2019) and mouse sperm (Jung *et al.*, 2017) showing enrichment of accessible DNA regions near gene promoters and enhancers, though a recent study has questioned whether the profiles in mouse sperm have been largely due to extracellular DNA contamination (Yin *et al.*, 2023). Another approach to label accessible DNA in intact cells is the so-called NicE techniques, in which a nicking endonuclease generates single-stranded DNA nicks in accessible regions of the genome that then serve as initiation sites for *E. coli* DNA Polymerase I to synthesize down-stream stretches of DNA (Ponnaluri *et al.*, 2017; Chin *et al.*, 2020, 2023). When biotinylated dNTPs are included with the polymerase, accessible DNA is labelled with biotin and can thus be captured by streptavidin and sequenced (NicE-seq; Chin *et al.*, 2023). NicE-seq has been reported to generate results similar to those seen using ATAC-seq, but with generally better resolution in fixed cells (Ponnaluri *et al.*, 2017). The NicE-view technique, in which fluorescently labelled nucleotides are incorporated by DNA Polymerase I downstream of DNA nicks induced by a nicking endonuclease allows visualization of accessible sites in single cells (Ponnaluri *et al.*, 2017; Chin *et al.*, 2020). To date, no studies using NicE-view in sperm have been reported.

Here, by using computational image analysis, we quantified levels of accessible DNA using NicE-view in millions of human spermatozoa from normozoospermic infertile individuals (and controls) with high blastocyst growth rate or no embryos developing after normal fertilization rates.

## Materials and methods

### Participant selection

This study was approved by the Ethikkommission Nordwest- und Zentralschweiz (EKNZ, BASEC-ID 2020-00330) and the design of the clinical trial was published (ClinicalTrials.gov ID NCT04256668). Sixty study participants were recruited from infertile men previously diagnosed and treated at the clinic of Reproductive Medicine and Gynecological Endocrinology (RME) of the University Hospital of Basel. Participants to the experimental study were selected based on the presence of a previously obtained conventional semen analysis showing values within the WHO reference range (Cooper *et al.*, 2010). Partners of selected individuals had normal antral follicle counts and AMH values within standard range (>10 pmol/l). Individuals in the naturally occurring pregnancy (NATP) group obtained a pregnancy following an initial semen analysis either spontaneously or following a non-ART treatment modality. Individuals in the high blastocyst growth rate (HBGR) and low blastocyst growth rate (LBGR) groups, respectively, were selected from those who underwent ART-based treatment at RME (either ICSI or IVF) between 2018 and 2021. In all individuals selected, the rate of generation of zygotes following ART was normal. Inclusion in the HBGR group was indicated by the production of blastocysts from at least 50% of cultured embryos, while LBGR group inclusion was defined by generation of at most one blastocyst following embryo culture. Between April 2020 and December 2023 participants fulfilling these criteria were contacted and asked to provide an additional semen sample for the study. All provided informed consent prior to providing semen samples for analysis.

Samples used for fluorescence-activated cell sorting (FACS) isolation of sperm based on CMA3 levels were obtained from participants from an additional cohort recruitment that had also been approved by the Ethikkommission Nordwest- und Zentralschweiz (EKNZ 2017-01407) and whose sperm were previously examined for alterations in the whole genome methylation (Stenz *et al.*, 2022). This cohort consisted of a control group of 10 fertile individuals who had previously donated sperm (and whose semen parameters were within the WHO standard ranges) and a group of 10 infertile individuals with normal semen parameters, but anogenital distance (AGD) of less than 40 mm (Eisenberg *et al.*, 2012) and with >20% terminal dUTP Nick End labelling (TUNEL)-positive swim-up sperm in a previous donation (Ribeiro *et al.*, 2017).

For ATAC-see, samples from five individuals were selected from the initial study cohort where sufficient total and swim-up material remained following the initial NicE-view staining. Two individuals each from the NATP and HBGR groups, and one from the LBGR were selected.

### Participant data and statistical analyses

Participant data were extracted from a clinic database and coded to remove all personally identifiable information with coding only available to clinical personnel (C.D.G.). Coded data were then entered into a secuTrial database (interActive Systems GmbH, Berlin Germany), which was accessible to researchers. Data were extracted from this database and analysed using R (R Core Team, 2021). Summary tables and statistical calculations were generated using the gt (Iannone *et al.*, 2020) and gtsummary (Sjoberg *et al.*, 2021) R packages.

### Semen sample collection and processing

Participants were requested to abstain from sexual activity for at least 2 days prior to providing a semen sample through masturbation at RME (actual abstinence times ranged from 1 to 14 days). Semen samples were allowed to liquefy at 37°C and then processed for conventional semen analysis in the andrology laboratory at RME (Cooper *et al.*, 2010). Semen quality assessment followed criteria established in Björndahl *et al.* (2022) with the following exceptions: Sperm concentration was assessed via computer-assisted sperm analysis and Sperm morphology was assessed following Diff-Quik staining rather than Papanicolaou staining. The complete checklist for semen quality analysis is included online as Supplementary Data File S1. A portion of each semen sample was subjected to a swim-up preparation to enrich for motile sperm (De Geyter *et al.*, 1988). In 57 of 60 cases, sufficient motile sperm was obtained for analysis, while swim-up preparation from three individuals resulted in insufficient material for further experimentation. For this study, total sperm refers to liquefied semen that was washed once in modified Ham’s F-10 (MHF-10) media. A fraction of total sperm was used for a TUNEL assay for DNA fragmentation (Ribeiro *et al.*, 2017). Total and swim-up sperm were dried onto 10-well diagnostic slides (VWR, Ranor, USA, No. 631-1371; 30000 sperm per well) and then transferred to −20°C for storage.

### NicE-view staining of human sperm samples

Ten-well slides of total and swim-up sperm were thawed at room temperature and washed once with phosphate-buffered saline (PBS, 50 µl per well). Spermatozoa were then fixed with 1% paraformaldehyde (Electron Microscopy Sciences, Hatfield, PA, USA, No. 15713) in PBS for 10 min at room temperature, followed by two 5-min washes in PBS. Sperm were permeabilized using Omni ATAC permeabilization buffer (10 mM Tris, pH 7.5, 10 mM NaCl, 3 mM MgCl_2_, 0.1% Igepal, 0.1% Tween-20, 0.01% digitonin) without DTT for 15 min at room temperature and then washed once with PBS (Corces *et al.*, 2017). For T4 NicE-view, following permeabilization samples were incubated for 15 min at room temperature with 30 µl per well of T4 DNA ligase (ThermoFisher Scientific, Waltham, MA, USA, No. EL0012) in 1× T4 DNA ligase buffer (40 mM Tris–HCl, 10 mM MgCl_2_, 10 mM DTT, 0.5 mM ATP, pH 7.8) and subsequently washed once with 50 µl per well PBS for 5 min. Samples were then labelled for 2 h at 37°C in 30 µl per well labelling mix. The NicE-view labelling mix was based on previously published protocols (Ponnaluri *et al.*, 2017; Chin *et al.*, 2020, 2023) and consisted of 1× NEBuffer 2 (NEB, Ipswich, MA, USA, No. B7002S), 30 µM dGTP (ThermoFisher Scientific, No. R0181), 30 µM dTTP (ThermoFisher Scientific, No. R0181), and 30 µM dmCTP (Jena Bioscience, Jena Germany, No. NU-1125L), 24 µM dATP (ThermoFisher Scientific, No. R0181), 6 µM Fluorescein-dATP (Jena Biosciences, No. NU-1611-FAMX), 3.2 U/ml Nt.CviPII (NEB No. R0626S), and 64 U/ml *E. coli* DNA Polymerase I (NEB No. M0209L). For Nick translation, the nicking endonuclease Nt.CviPII was excluded, while for negative control reactions both Nt.CviPII and DNA Polymerase I were excluded. Following labelling, samples were washed 3 times with PBS+0.01% SDS+50 mM EDTA, pre-heated to 55°C, for 15 min each. Samples were counter-stained with DAPI (10 µg/ml in PBS, Sigma Aldrich, St Louis, MO, USA, No. D9542-10MG) for 5 min at room temperature, mounted with VectaShield (Vector Laboratories, Newark, NJ, USA, No. H-1000) and covered with 50 mm coverslips. Slides were then sealed with nail polish. Slides were stored at 4°C in the dark until imaging (maximally 2 days).

### Double labelling of DNA nicks and NicE-view signal on human sperm

For double labelling, sperm were processed as for standard NicE-view labelling with the following exceptions. Following fixation and permeabilization, endogenous DNA nicks were labelled using Fluorescein-labelled dATP (as above). Ends of newly synthesized DNA strands were then sealed by incubating with T4 DNA ligase (ThermoFisher Scientific, EL0012) in 1× T4 DNA ligase buffer overnight at room temperature in a humid chamber in the dark. A second-labelling reaction with ATTO565-labelled dATP (Jena Biosciences, No. NU-1611-565) was then performed either with Nt.CviPII or without (as control) in the dark. Post-labelling processing was as in standard NicE-view labelling (described above).

### FACS sorting of sperm based on CMA3 levels and microscopic analysis

Sperm from fertile participants and infertile participants with decreased anogenital distance and increased percentages of TUNEL positive was subjected to sorting based on CMA3 levels as previously described (Stenz *et al.*, 2022). Briefly, total media-washed sperm were incubated in the dark for 30 min at room temperature with 0.25 mg/ml CMA3 (Sigma Aldrich, No. C2659-10MG) and 5 µM Vybrant DyeCycle Ruby (ThermoFisher Scientific, No. V10273) diluted in McIlvaine’s buffer, pH 7.0, supplemented with 10 mM MgCl_2_. Sperm were then washed once with diluent. FACS was performed using a BD Influx (BD Biosciences, Franklin Lakes, NJ, USA) equipped with 355, 488 and 640 nm lasers. Cells were first gated using FSC and SSC to eliminate debris. Haploid sperm were then further selected from based on DNA content (using Vybrant DyeCycle Ruby). Finally, the selected sperm were sub-divided into two populations based on CMA3 levels. These selected sperm were then dried onto 10-well diagnostic slides and stored at −20°C. Slides were later subjected to T4 NicE-view as described above.

### ATAC-see staining of human sperm

Oligonucleotides for ATAC labelling were synthesized and HPLC purified by Microsynth AG (Balgach, Switzerland). The two forward oligos (TCGTCGGCAGCGTCAGATGTGTATAAGAGACAG and GTCTCGTGGGCTCGGAGATGTGTATAAGAGACAG) were labelled at their 5′ ends with ATTO488 (ATTO-Tec Siegen Germany), while a common reverse oligo (CTGTCTCTTATACACATCT) was 5′ phosphorylated. Forward and reverse oligos were annealed at a concentration of 1.5 µM in annealing buffer (10 mM Tris pH 7.5, 50 mM NaCl and 1 mM EDTA) by heating to 95°C for 5 min and subsequently cooling to 25°C over a period of 45 min in a thermocycler. Recombinant ZZ-tagged Tn5 (Fanourgakis *et al.*, 2025) was purified from *E. coli*. Tn5 (∼5 µM) was loaded with labelled annealed oligos (6 µM) by mixing and incubating at 37°C for 30 min. Equimolar volumes of Tn5 loaded with each of the two different annealed oligos were pooled and stored at −20°C until use. Pre-processing of 10-well slides was the same as for NicE-view (up to permeabilization). Transposition reactions were performed in 1× TDM buffer (10 mM Tris, pH 7.5, 10% DMF, 5 mM MgCl_2_). Fluorescent oligo-loaded Tn5 was diluted 1:100 for use. For the no Magnesium control, 5 mM MgCl_2_ was replaced by 5 mM EDTA. Transposition reactions were performed at 37°C for 1 h in a humid chamber in the dark. Post-staining processing (washing, counter-staining, and mounting) was the same as for NicE-view.

### NicE-view and ATAC-see imaging

Stained slides were imaged on a Visitron Spinning Disk W1 confocal microscope (Visitron Systems GmbH, Puchheim, Germany). Images were acquired with a 40× oil objective (Zeiss, NA: 1.3, WD 0.21) using a 405-nm laser with a 460/50 filter for DAPI and a 488-nm laser with a 525/50 filter for NicE-view and ATAC signal using a PCO.edge 4.2M sCMOS camera (PCO Imaging, Kelheim, Germany). For double-labelling experiments, a 561-nm laser with a 609/54 filter was also utilized. For each well, two separate 5 × 5 tiles were acquired including a 6-µm Z-stack for each image (with 1 µm step size). For Nick translation, NicE-view and T4 NicE-view, 2-wells per slide were independently stained resulting in 100 image stacks per slide for these assays. For negative control and T4 DNA ligase treatment of Nick translation, one well per slide was stained resulting in 50 image stacks per slide for these controls.

### NicE-view and ATAC-see image processing

**Figure 1.**
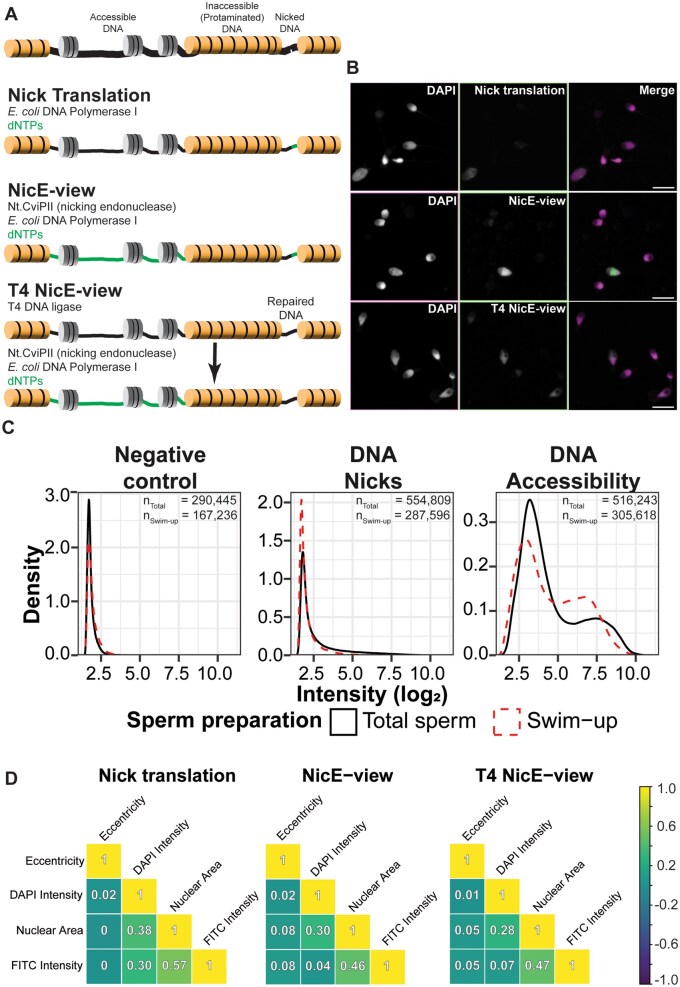
**NicE-view reveals variation between DNA accessibility in individual human sperm.** (**A**) Cartoon showing different staining conditions used to measure presence of accessible DNA and nicked DNA in sperm samples. (**B**) Example images showing results of staining outlined in (A). The right panels show merged images from the two left panels (with DAPI signal in magenta and the DNA labelling signal in green). Brightness and contrast for Nick translation image were enhanced relative to the (T4) Nice-view images to make signal more visible. The corresponding unenhanced image is shown in Supplementary Fig. S1. Scale bars = 10 μm. (**C**) Density plot showing distributions of log2 transformed integrated intensities for sperm stained with conditions indicated in (A). Total sperm numbers per condition are indicated. (**D**) Correlation plots showing Spearman’s ρ for a set of selected features measured in all (total and swim-up) sperm samples.

Maximum intensity projections were generated for each stack in VisiView (Visitron Systems, Puchheim Germany). Projections were processed with Fiji (Schindelin *et al.*, 2012) and Photoshop (Adobe Systems, San Jose, CA, USA) to generate images shown in Fig. 1B and Supplementary Fig. S1B. Projections were further processed using CellProfiler (Carpenter *et al.*, 2006; Stirling *et al.*, 2021). Nuclear segmentation was performed on DAPI images (Segmentation Parameters: 25–50-pixel diameter, Adaptive Sauvola thresholding with no smoothing scale, a threshold correction factor of 1.0, and an adaptive window size of 800). Clumped objects were distinguished by shape with intensity used to draw dividing lines between clumped objects. Segmentation masks were inspected visually to confirm reasonable segmentation. Intensity values and size and shape parameters were then calculated for each segmented nucleus. The parameters measured and examined were defined by CellProfiler (Carpenter *et al.*, 2006; Stirling *et al.*, 2021) as: Integrated Intensity: The sum of the pixel intensities within an object. Mean Intensity: The average pixel intensity within an object. Std. Dev. Intensity: The standard deviation of the pixel intensities within an object. Max Intensity: The maximal pixel intensity within an object. Minimum Intensity: The minimal pixel intensity within an object. Integrated Edge Intensity: The sum of the edge pixel intensities of an object. Mean Edge Intensity: The average edge pixel intensity of an object. Std. Dev. Edge Intensity: The standard deviation of the edge pixel intensities of an object. Max Edge Intensity: The maximal edge pixel intensity of an object. Minimum Edge Intensity: The minimal edge pixel intensity of an object. Mass Displacement: The distance between the centres of gravity in the grey-level representation of the object and the binary representation of the object. Lower Quartile Intensity: The intensity value of the pixel for which 25% of the pixels in the object have lower values. Median Intensity: The median intensity value within the object. Median absolute deviation (MAD) Intensity: The MAD value of the intensities within the object. The MAD is defined as the median(|*x_i_*−median(*x*)|). Upper Quartile Intensity: The intensity value of the pixel for which 75% of the pixels in the object have lower values. Area: The number of pixels in the region. Perimeter: The total number of pixels around the boundary of each region in the image. Form Factor: Calculated as 4 × π × Area/Perimeter^2^. Equals 1 for a perfectly circular object. Solidity: The proportion of the pixels in the convex hull that are also in the object, i.e. Object Area/Convex Hull Area. Extent: The proportion of the pixels in the bounding box that are also in the region. Computed as the area/volume of the object divided by the area/volume of the bounding box. Bounding Box Area: The area of a box containing the object. Eccentricity: The eccentricity of the ellipse that has the same second moments as the region. The eccentricity is the ratio of the distance between the foci of the ellipse and its major axis length. The value is between 0 and 1 (0 and 1 are degenerate cases; an ellipse whose eccentricity is 0 is actually a circle, while an ellipse whose eccentricity is 1 is a line segment.). Major Axis Length: The length (in pixels) of the major axis of the ellipse that has the same normalized second central moments as the region Min Axis Length: The length (in pixels) of the minor axis of the ellipse that has the same normalized second central moments as the region. Equivalent Diameter: The diameter of a circle with the same area as the object. Compactness: The mean squared distance of the object’s pixels from the centroid divided by the area. A filled circle will have a compactness of 1, with irregular objects or objects with holes having a value greater than 1. Maximum Radius: The maximum distance of any pixel in the object to the closest pixel outside of the object. For skinny objects, this is 1/2 of the maximum width of the object. Median Radius: The median distance of any pixel in the object to the closest pixel outside of the object. Mean Radius: The mean distance of any pixel in the object to the closest pixel outside of the object. Minimum Feret Diameter, Maximum Feret Diameter: The Feret diameter is the distance between two parallel lines tangent on either side of the object (imagine taking a calliper and measuring the object at various angles). The minimum and maximum Feret diameters are the smallest and largest possible diameters, rotating the callipers along all possible angles.

### Imaging data analysis

All data analyses were performed in R. Comma-separated value (csv) files generated by CellProfiler were loaded into R and pre-processed using the tidyverse suite of packages (Wickham, 2016; Wickham *et al.*, 2019). Correlation matrices and their significance values were generated using the rstatix package (cor_mat parameters): method = ‘spearman’, alternative = ‘two.sided’, adjust_pvalue (method = ‘fdr’) (Kassambara, 2023b). Thresholding of Nick translation, NicE-view, and T4 NicE-view distributions was performed as follows. A kernel density estimation was performed for log_2_-transformed signal intensity values for each sample and staining condition (in a range of median ±2). Optimization was performed on an approximated function derived from this estimation and the minimum value from this optimization was determined. For NicE-view and T4 NicE-view, this value was used as the threshold to separate sperm into highly or lowly stained categories. For Nick translation, the value calculated for the Negative control of the same sample was used as the threshold value.

### Data visualization

**Figure 2.**
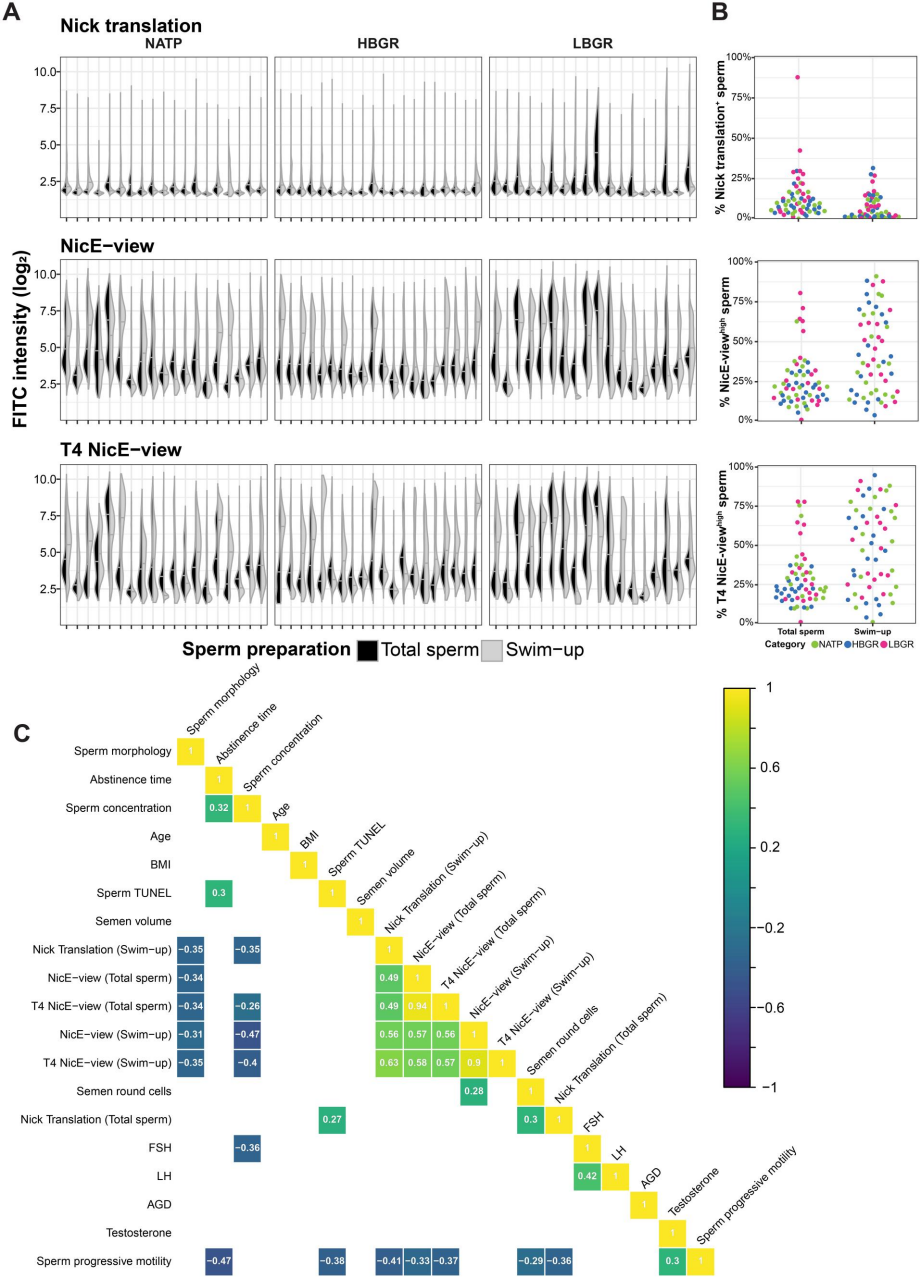
**Frequency of sperm with differing levels of DNA accessibility varies between individuals and preparation methods.** (**A**) Split violin plots showing the distribution of single sperm integrated intensity values from 57 individuals stained with Nick translation, NicE-view and T4 NicE-view. Black distributions (left) represent total sperm, while grey distributions (right) refer to sperm selected for motility via swim-up preparation. (**B**) Beeswarm plots showing distributions of frequencies of highly labelled sperm (for each assay) obtained from thresholding distributions seen in (A), coloured by participant category. For total sperm n = 60, for swim-up n = 57. (**C**) Correlation plot showing Spearman’s ρ for a set of participant-level features. Values are only displayed for correlations with an FDR-corrected *P*-value of <0.05.

**Figure 3.**
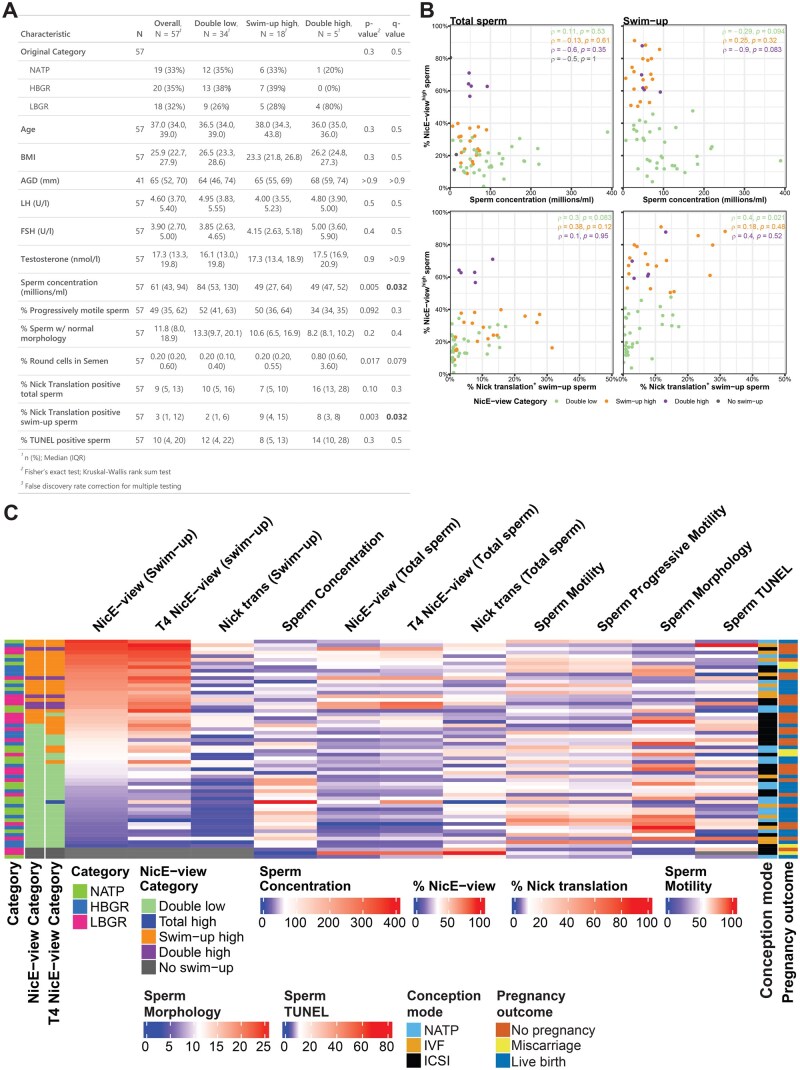
**Sperm concentration is negatively correlated with the frequency of sperm with high levels of DNA accessibility.** (**A**) Table showing breakdown of participant parameters following re-categorization based on NicE-view frequencies (from Fig. 2B). Data from 57 individuals possessing both total and swim-up samples are shown. Double low: <50% of sperm with high DNA accessibility in both total and swim-up sample. Double high: ≥50% of sperm with high accessibility in both total and swim-up sample. Swim-up high: ≥50% of sperm with high accessibility in swim-up sample, but not in total sample. For numerical variables, a Kruskal–Wallis rank sum test was used to compare groups, while for Original Category a Fisher’s exact test was used. Statistical comparisons of different parameters were corrected for multiple hypothesis testing using a false discovery rate calculation (listed as ‘qvalue’ in table); values in bold are statistically significant. (**B**) Scatter plots comparing Sperm concentration or the % Nick translation^+^ swim-up sperm to the % NicE-view^high^ sperm from total (left) or swim-up (right) sperm. Points are coloured based on NicE-view Categories as defined in (A). Values shown are Spearman’s ρ by category. (**C**) Heatmap showing relationship between spermatological parameters, DNA accessibility, and DNA nicking in all samples examined. Data are ordered by % NicE-view^high^ sperm in swim-up sample.

**Figure 4.**
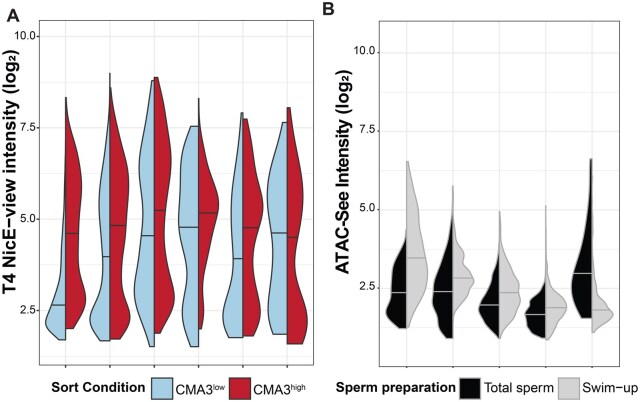
**NicE-view intensity barely correlates with chromomycin A3 (CMA3) and provides better separation of lowly and highly labelled sperm than ATAC-see.** (**A**) Split violin plot showing the distribution of log_2_ integrated T4 NicE-view intensities from individual sperm from samples fractionated by FACS according to CMA3 staining intensity from six individuals. Sperm with low CMA3 levels are shown in blue on left, while sperm with high CMA3 levels are shown in red on right. The first and third plots from the left are derived from infertile individuals with increased anogenital distance, while the others are from fertile males. (**B**) Split violin plot showing the distribution of log_2_ integrated ATAC-see intensities from individual sperm from total and swim-up preparations from five individuals.

**Figure 5.**
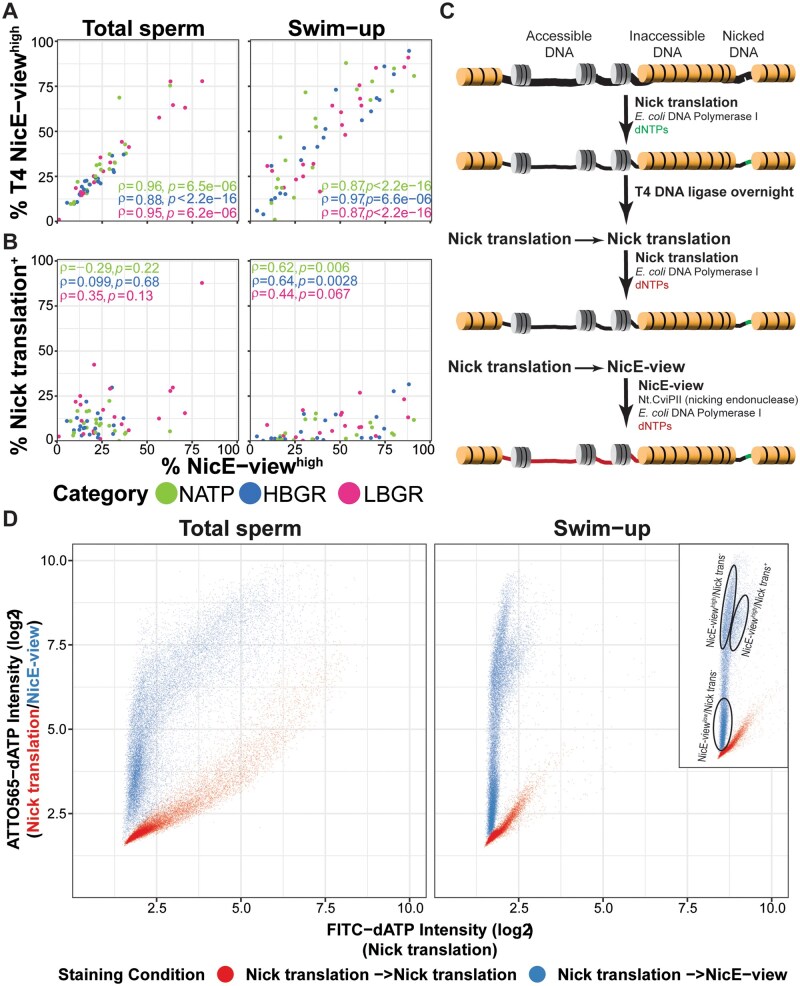
**DNA accessibility and DNA nicking correlate in human sperm samples.** (**A, B**) Scatter plot showing relationship between % NicE-view^high^ and % T4 NicE-view^high^ (A) or % Nick translation^+^ (B) sperm in total (left) and swim-up (right) samples. Points are coloured by participant category. Values presented are Spearman’s ρ per category. (**C**) Cartoon describing double-labelling experiment to label DNA nicks and accessible regions in the same cell. (**D**) Scatter plot showing the results of double-labelling experiments. Red points indicate Nick translation followed by Nick translation, while blue points indicate Nick translation followed by NicE-view. For both samples, value on *y*-axis represents Nick translation level. For red points, value on *x*-axis represents incomplete DNA repair by T4 DNA ligase (likely from highly damaged sperm), while for blue points value on *x*-axis represents NicE-view signal. Inset shows subpopulations identifiable in scatter plot. Data presented represent a pool of samples from six individuals.

Most plots were generated using ggplot2 (Wickham *et al.*, 2019). The ggpubr package was used to add correlation coefficients to scatter plots (Kassambara, 2023a). Correlation plots (Figs 1D and 2C, Supplementary Fig. S2) were generated using the cor_plot() function from the rstatix package (Kassambara, 2023b). Colours were generated using the viridis package (Garnier *et al.*, 2024). Beeswarm plots (Fig. 2B) were generated using the ggbeeswarm package (Clarke *et al.*, 2023). Plots were combined using the patchwork package (Pedersen, 2024). For plots showing distributions of values between individuals, 1000 sperm were randomly sub-sampled (using sample_n(); Wickham *et al.*, 2019) from each sperm sample (total and swim-up) to generate distributions equally weighted for each individual. For Fig. 2C, only values with an FDR-corrected *P* < 0.05 were displayed. The table (Fig. 3A) showing statistical significance of parameters upon re-categorization by NicE-view behaviour was generated using the gtsummary package (Sjoberg *et al.*, 2021). Heatmaps (Fig. 3C, Supplementary Fig. S3) were generated by the ComplexHeatmap package (Gu *et al.*, 2016; Gu and Hubschmann, 2022). Colour scales for each numerical variable were generated by the colorRamp2() function from the circlize package (Gu *et al.*, 2014) such that median values were coloured white, while minimum values were blue and maximum values red. Split violin plots (Figs 2A and 4) were generated using custom code for ggplot2. Row order for heatmaps was determined by the frequency of NicE-view^high^ swim-up sperm. Scatter plot shown in Fig. 5D was generated using the scattermore package (Kulichova and Kratochvil, 2023).

## Results

### Selection of individuals with sperm of differing effects on embryonic competence

Comparison of features between participant groups showed no significant differences in age, BMI, or AGD (Table 1). Analysis of age, BMI, endocrine features, and antral follicle counts in partners of study participants showed no significant differences between groups, minimizing the possibility for ovarian reserve or oocyte-derived factors in driving differences in pre-implantation embryo development rates in these couples (Table 1). Hormonal parameters (LH, FSH, and testosterone) were also not significantly different between groups (Table 1). Comparison of semen parameters between groups showed a significant decrease in the percent of progressively motile sperm in the LBGR group relative to the two others, but no differences in any other parameters (Table 1). Despite this, the semen samples (acquired at time of recruitment) of 15 of 20 LBGR individuals possessed progressive motility values above the 5th percentile reference values for fertile individuals as determined by the WHO (Cooper *et al.*, 2010). The presence of slightly decreased sperm motility in the LBGR group led clinicians at the time of treatment decision to opt for ICSI as opposed to IVF more frequently in this subgroup.

**Table 1.** Characteristics of primary participant cohort.

Characteristic	N	**Overall, N = 60** ^1^	**NATP, N = 20** ^1^	**HBGR, N = 20** ^1^	**LBGR, N = 20** ^1^	***P*-value** ^2^	***q*-value** ^3^
**Age**	60	37.0 (34.0–40.0)	35.0 (32.8–37.0)	38.0 (34.8–40.8)	38.0 (36.0–40.5)	0.043	0.12
**BMI**	60	25.9 (22.8–27.8)	24.2 (22.3–26.2)	25.9 (21.8–28.1)	27.3 (24.8–29.1)	0.037	0.12
**Anogenital distance (mm)**	43	65 (51–70)	64 (52–67)	64 (53–69)	68 (49–73)	0.87	0.91
**LH (U/l)**	60	4.70 (3.78–5.63)	4.05 (3.70–5.23)	4.95 (3.98–5.33)	4.90 (3.58–6.73)	0.55	0.77
**FSH (U/l)**	60	4.05 (2.70–5.03)	3.75 (2.93–5.13)	3.85 (2.48–4.55)	4.65 (3.08–5.53)	0.51	0.75
**Testosterone (nmol/l)**	60	17.1 (13.1–19.8)	15.4 (13.5–21.2)	17.7 (13.6–19.8)	15.2 (12.4–18.3)	0.26	0.45
**Sperm concentration (millions/ml)** **(Historical sample)**	60	67 (36–98)	61 (36–98)	86 (50–103)	51 (21–88)	0.13	0.27
**% Progressively motile sperm** **(Historical sample)**	60	52 (38–64)	60 (46–64)	61 (48–75)	40 (33–47)	0.001	**0.012**
**% Sperm w/normal morphology** **(Historical sample)**	59	11.0 (7.0–16.0)	12.0 (8.5–15.5)	11.0 (7.8–15.3)	8.5 (6.0–18.3)	0.63	0.85
**Sperm concentration (millions/ml)** **(Study sample)**	60	60 (37–93)	64 (41–93)	68 (54–108)	48 (27–76)	0.16	0.33
**% Progressively motile sperm** **(Study sample)**	60	49 (34–62)	52 (49–63)	61 (42–66)	35 (26–44)	<0.001	**0.009**
**% Sperm w/normal morphology** **(Study sample)**	60	11.3 (7.1–18.5)	12.2 (7.7–14.9)	11.3 (9.8–18.5)	9.6 (6.2–19.6)	0.80	0.88
**% TUNEL positive sperm** **(Study sample)**	59	10 (5–20)	7 (5–16)	9 (4–16)	19 (9–30)	0.060	0.15
**% Round cells in Semen**	60	0.20 (0.18–0.60)	0.20 (0.18–0.40)	0.20 (0.10–0.23)	0.60 (0.20–3.38)	0.017	0.10
**Semen volume (ml)**	60	2.60 (1.93–3.85)	2.80 (2.00–4.28)	2.25 (1.58–3.05)	2.60 (1.93–3.53)	0.71	0.86
**Age of partner**	60	35.0 (33.0–37.0)	34.0 (30.8–35.3)	35.0 (33.0–36.3)	36.0 (34.0–38.3)	0.037	0.12
**BMI of partner**	60	22.8 (20.7–26.6)	21.2 (19.6–23.8)	23.9 (20.8–26.8)	24.2 (22.1–27.5)	0.034	0.12
**Partner d3 E2 (pmol/l)**	59	139 (110–192)	146 (105–248)	138 (124–168)	136 (94–180)	0.82	0.88
**Partner d3 FSH (U/l)**	59	6.70 (5.30–7.75)	6.30 (4.80–6.90)	6.70 (5.43–7.50)	7.15 (6.08–8.28)	0.072	0.17
**Partner d3 LH (U/l)**	59	6.40 (5.10–7.80)	6.80 (4.45–7.85)	6.35 (5.35–7.73)	6.35 (5.10–7.90)	0.94	0.94
**Partner d3 AMH (pmol/l)**	59	20 (13–30)	21 (12–36)	22 (16–32)	18 (10–24)	0.29	0.45
**Partner d3 Prolactin (mIU/l)**	59	275 (204–428)	238 (180–355)	276 (222–457)	304 (235–432)	0.25	0.45
**Partner d3 TSH (mIU/l)**	60	1.97 (1.48–2.46)	1.83 (1.48–2.33)	2.14 (1.55–2.77)	2.05 (1.58–2.51)	0.71	0.86
**Partner d3 TPO Ab (IU/l)**	60	15 (15–15)	15 (15–16)	15 (15–15)	15 (15–22)	0.75	0.88
**Partner antral follicle count**	60	20 (17–30)	24 (18–37)	20 (17–24)	19 (16–25)	0.27	0.45
**ART type**	40					0.041	0.12
ICSI		27 (68)	0 (NA)	10 (50)	17 (85)		
IVF		13 (33)	0 (NA)	10 (50)	3 (15)		
**Pregnancies**	60	34 (57)	20 (100)	12 (60)	2 (10)	<0.001	**<0.001**
**Pregnancy outcome**	34					0.018	0.10
Delivery		28 (82)	18 (90)	10 (83)	0 (0)		
Miscarriage		6 (18)	2 (10)	2 (17)	2 (100)		

LBGR, low blastocyst growth rate; HBGR, high blastocyst growth rate.

1 Median (IQR); n (%); NA, not applicable.

2 Kruskal–Wallis rank sum test; Fisher’s exact test.

3 False discovery rate correction for multiple testing, values in bold are statistically significant.

### NicE-view reveals two primary subpopulations of sperm in human samples

To study the chromatin state in the nuclei of spermatozoa, we modified the NicE-view assay to label accessible DNA in frozen dried sperm samples. This assay introduces single-stranded DNA nicks into regions of accessible chromatin and then labels areas surrounding these nicks via introduction of labelled nucleotides by *E. coli* DNA polymerase I (Fig. 1A). We first optimized permeabilization of the sperm, using a modified version of the permeabilization buffer developed for Omni ATAC-seq, which has been shown to work in a variety of fixed cells (Corces *et al.*, 2017). For use in sperm, we removed DTT from this buffer, to prevent reduction of disulphide bonds in protamines and possible alteration of chromatin structure in these cells. NicE-view cannot distinguish between endogenous single-stranded DNA nicks and accessible chromatin. It has previously been reported that some human sperm contain substantial levels of endogenous DNA nicks (Bianchi *et al.*, 1993; Manicardi *et al.*, 1995), a result we confirmed by examining DNA nicking using Nick translation (Fig. 1A and B).

We therefore modified NicE-view to more specifically label only accessible chromatin by treating samples with T4 DNA ligase prior to NicE-view labelling (referred to as T4 NicE-view). We evaluated whether this pre-treatment was effective at repairing DNA nicks in this context by treating samples with T4 DNA ligase prior to performing a Nick translation assay (referred to as T4 Nick translation, Supplementary Fig. S1A and B). We found that this pre-treatment was indeed effective at suppressing signals from Nick translation, though this suppression was less efficient in samples with exceedingly high signal, where DNA is likely more highly fragmented (Fig. 1C). In brief:

- Nick translation reflects the quantity of endogenous DNA nicks
- NicE-view reflects accessible portions of the genome and regions possessing endogenous DNA nicks
- T4 NicE-view reflects only accessible portions of the genome.

Imaging of human sperm with the modified NicE-view protocols (with and without T4 DNA ligase) revealed a wide variation in signal intensities between individual spermatozoa within each sample (Fig. 1A). Generally, signal intensity for the NicE-view (and T4 NicE-view) labelled samples was much higher than that seen with Nick translation (Fig. 1B, Supplementary Fig. S1B). We imaged sperm labelled via Nick translation, NicE-view, and T4 NicE-view (and controls) from 117 samples derived from 60 individuals by confocal microscopy. These included 60 total washed semen samples (referred to as total sperm) and 57 sperm samples prepared using the swim-up method to select for motile sperm.

To obtain a quantitative view of DNA labelling in individual spermatozoa, we performed segmentation and quantification of nuclear signal from these images leading to measurements of 3 376 931 individual spermatozoa. Plotting the density of signal intensities across different staining conditions showed that negative controls (treated with fluorescently labelled dNTPs but no enzymes) possessed very low background signals, while Nick translation signal was similar in intensity to background levels for the majority of sperm, with only a small population showing a relatively high signal (Fig. 1C). Spermatozoa with a high Nick translation signal were more abundant in total sperm when compared to swim-up prepared sperm (Fig. 1C), consistent with previous studies showing that swim-up selection decreases the frequency of DNA fragmentation in sperm (Younglai *et al.*, 2001; Oguz *et al.*, 2018). T4 DNA ligase pretreatment reduced the intensity of the Nick translation signals but did not fully suppress the high Nick translation signal observed in brightly labelled sperm: This was more clearly seen in total sperm (Fig. 1C), suggesting that within the total sperm population, a group of sperm with high levels of DNA nicks is present that cannot be repaired by T4 DNA ligase treatment and is mostly removed via selection for sperm motility.

In contrast to Nick translation, where most sperm showed signal near to background, the vast majority of sperm stained via NicE-view and T4 NicE-view showed clear labelling (Fig. 1C). We observed a well-delineated bimodal distribution of signal intensities in both of these assays, an effect present in both total and swim-up sperm (Fig. 1C). We also observed that the values for the two peaks of signal intensities for swim-up sperm were slightly decreased relative to total sperm in both assays, though the frequency of sperm in the upper portion of the distribution was increased in swim-up prepared samples (Fig. 1C).

Next to measuring levels of endogenous nicks and chromatin accessibility across whole sperm nuclei, represented by the integrated FITC intensity, we also quantified a variety of features related to DNA labelling, such as DAPI staining intensity and the size/shape of the nuclei of the spermatozoa. Correlation analysis of many of these features showed strong correlations as they are highly interdependent variables (Supplementary Fig. S2). We selected three labelling/staining approaches demonstrating four of the major independent features measured in this study: (i) Nuclear area, (ii) Eccentricity (representing the level of nuclear elongation), (iii) integrated DAPI intensity (representing total DNA staining levels), and (iv) FITC Intensity. Analysis of these four variables across all measured sperm showed a clear correlation between Nuclear area and DAPI intensity (regardless of assay) (Fig. 1D). We also observed correlations between the extent of enzymatic DNA labelling (i.e. FITC intensity) and nuclear area for all three staining approaches (Fig. 1D). DAPI intensity and the extent of enzymatic DNA labelling showed a variable effect based on staining condition: while signal intensity derived from endogenous nicks showed a correlation with total DNA staining level, accessible DNA labelling showed no such effect (Fig. 1D). None of the selected variables showed a correlation with nuclear shape as measured by eccentricity (Fig. 1D).

### DNA nicking and NicE-view labelling vary between individuals and correlate with reproductive parameters

We next asked how the distribution of NicE-view signal intensities varied between individual study participants. This analysis showed a wide variety of values across participants (Fig. 2A). We observed a few patients of the LBGR sub-group with relatively high levels of endogenous DNA nicking in their total sperm, which were largely absent following selection via swim-up (Fig. 2A). There was also a tendency towards increased frequencies of highly accessible DNA in total spermatozoa of individuals from the LBGR sub-group compared to the others (Fig. 2A). Remarkably, we observed a tendency towards higher frequencies of sperm with increased DNA accessibility, measured both with NicE-view and T4 NicE-view, in swim-up samples from all cohort sub-groups (Fig. 2A).

To compare values from our labelling assays to other reproductive parameters, we utilized a thresholding approach to sub-divide sperm into groups based on signal intensity. To threshold Nick translation signal, we identified a non-zero local minima in our Negative Control distributions for each sample using a kernel density function estimation (see ‘Imaging data analysis’ in Materials and Methods for more details) and applied these as cutoff values defining sperm with greater than these values as Nick translation positive (Supplementary Fig. S3A). For NicE-view and T4 NicE-view, we used a kernel density function estimation to identify the local minimum around the median signal intensity value for each distribution in each sample. We defined sperm with values above the local minima as NicE-view^high^ and those with values below as NicE-view^low^ (Supplementary Fig. S3B). This thresholding clarified the trends shown in Fig. 2A. The number of total sperm with DNA nicks was clearly higher than that seen in swim-up selected sperm, with a subset of LBGR individuals showing higher values in total sperm relative to the other participants (Fig. 2B). The distribution of NicE-view^high^ and T4 NicE-view^high^ sperm frequencies in total sperm show that only ∼10% samples possessed greater than 50% NicE-view^high^ sperm (Fig. 2B). Among the individuals with greater than 50% NicE-view^high^ in total sperm, five out of six were LBGR and for T4 NicE-view this was five out of seven (Fig. 2B). In contrast, following swim-up selection, ∼40% of all individuals had frequencies of NicE-view^high^ sperm greater than 50% (Fig. 2B). Unlike for total sperm samples, we observed no difference in the distributions of high- and low-frequency samples among the three different participant categories (Fig. 2B).

We next determined the correlation between the frequencies of DNA-nicked or DNA-accessible sperm and other reproductive parameters (Fig. 2C). We found strong correlations between NicE-view^high^ and T4 NicE-view^high^ frequencies in both total and swim-up sperm (Fig. 2C; ρ = 0.94 for total sperm; ρ = 0.9 for swim-up sperm). We also found that the frequency of swim-up sperm with DNA nicks, but not total sperm, positively correlated with DNA accessibility (Fig. 2C, ρ = 0.56 for Nick translation in swim-up sperm with NicE-view and ρ = 0.63 for Nick translation and T4 NicE-view in swim-up sperm). The frequency of Nick translation-positive total sperm showed a positive correlation with an independent measure of DNA fragmentation (TUNEL, which measures both single and double-stranded DNA damage; Ribeiro *et al.*, 2017), but this correlation disappeared in swim-up selected sperm (Fig. 2C, ρ = 0.27 for total sperm). Finally, we found that sperm parameters measured as part of conventional semen analysis like sperm morphology, progressive motility, and concentration are negatively correlated with the frequency of high DNA accessibility sperm across all samples (Fig. 2C).

### Re-categorization of participants based on DNA accessibility

Our measurements of high DNA accessibility frequencies in total and swim-up sperm indicated a bimodal distribution of values (Fig. 2B). Using a frequency of 50% NicE-view^high^ as a cutoff, we re-categorized our participants based on the behaviour of their sperm in both preparation methods into three categories: Double low (where >50% of sperm in both total and swim-up samples were NicE-view^low^), Double high (where ≥50% of sperm in both total and swim-up samples were NicE-view^high^), and Swim-up high (where ≥50% of swim-up sperm but <50% of total sperm were NicE-view^high^). About 59.6% of individuals were categorized as Double low, 8.8% of individuals as Double high, and 31.6% of individuals as Swim-up high (Fig. 3A).

Comparing our newly defined categories to those used for recruitment, we found that four out of five NicE-view Double high participants (80%) were LBGR. This enrichment is, however, not statistically significant owing to the low number of Double high individuals identified (Fig. 3A). Comparing our NicE-view-defined categories to other fertility parameters, we found that sperm concentration inversely correlates with NicE-view frequency (Fig. 3A). Comparing all individuals, no individuals from the Double high or Swim-up high categories had sperm concentrations greater than 100 million/ml, while 38% of individuals from the Double low category possessed a value above this level (Fig. 3B).

Moreover, we observed that the % Nick translation positive swim-up sperm correlated positively with NicE-view frequencies. For example, 50% of Swim-up high individuals had greater than 10% Nick translation positive swim-up sperm whereas only 20% of Double low individuals had swim-up Nick translation frequencies above this value (Fig. 3B). These data may point to a possible mechanistic connection between levels of intrinsic DNA damage and general chromatin accessibility in sperm of such individuals.

Finally, to compare all parameters and categories to each other, we plotted these data as a heatmap (Fig. 3C). The relationships between NicE-view, T4 NicE-view, and Nick translation values from swim-up sperm were clear, particularly for those with low frequencies for all parameters (Fig. 3C). The negative relationship between Swim-up NicE-view^high^ frequency and Sperm concentration was also evident (Fig. 3C). We did not observe an obvious relationship between any other measured parameters, or a further sub-grouping of participants based on these parameters (Fig. 3C, Supplementary Fig. S4).

### Relationship between NicE-view and other methods for detecting DNA accessibility

Previous studies reported variation in the staining level of the DNA dye CMA3 between sperm from the same sample (Bianchi *et al.*, 1993). This variation has been suggested to represent variation in protamine levels between sperm (Bianchi *et al.*, 1993) and has been correlated with poor reproductive outcomes (Ni *et al.*, 2016). To determine if NicE-view^high^ sperm and CMA3^high^ sperm represent the same subpopulation, we performed FACS on sperm stained with CMA3 to obtain CMA3^low^ and CMA3^high^ enriched sperm populations from four fertile and two infertile individuals (Stenz *et al.*, 2022). We then performed the T4 NicE-view assay on these two CMA3 populations. We found an overall positive correlation between the two assays. Five of six individuals show increased median T4 NicE-view signal intensities in CMA3^high^ compared to CMA3^low^ subpopulations (Fig. 4A). However, the presence of clear bimodal T4 NicE-view intensity distributions in both CMA3 subpopulations suggests that Nice-view measurements of chromatin accessibility are distinct from DNA labelling of sperm with the fluorescent CMA3 dye (Fig. 4A).

ATAC has been used extensively to measure DNA accessibility in many cell types including sperm (Buenrostro *et al.*, 2013; Chen *et al.*, 2016; Jung *et al.*, 2017, 2019). We utilized ATAC-see, a fluorescence microscopy-based variation of the ATAC labelling protocol (Chen *et al.*, 2016) to measure DNA accessibility in human sperm. We first tested whether incorporation of fluorescently labelled oligonucleotides into chromatin could occur in fixed sperm samples. We performed two control reactions to test for incorporation. First, we excluded Tn5 transposase, which showed the amount of fluorescence exclusively obtained from unincorporated labelled oligonucleotides. Secondly, we included Tn5 but excluded magnesium which is a cofactor necessary for Tn5’s transposition activity (Supplementary Fig. S5). Comparing these controls to ATAC-see reactions, we observed a moderately increased signal intensity in ATAC-see showing that enzymatic incorporation of oligonucleotides did occur in fixed human sperm samples. The level of signal enrichment compared to the negative controls was mild. We next examined the levels of ATAC-see signal in total and swim-up sperm from five individuals and observed that four of five individuals showed increased median signal in swim-up compared to total sperm (Fig. 4B). In contrast to NicE-view and T4 NicE-view, however, we did not observe a bimodal distribution of signals and overall signal intensities were low (Fig. 4B).

### Some, but not all, sperm with high DNA accessibility contain DNA nicks

Given the positive correlation between NicE-view^high^ and swim-up Nick translation frequencies (Figs 2C and 3A), we next asked whether this correlation was driven by a common subpopulation of sperm. Examination of individual samples showed a very strong correlation between frequencies of both NicE-view^high^ and T4 NicE-view^high^ sperm in both total and swim-up samples (Fig. 5A). In contrast, no correlation between the frequency of NicE-view^high^ sperm and Nick translation positive sperm was found in total sperm, while a positive correlation was found for NATP and HBGR individuals in swim-up samples (Fig. 5B).

To more directly assess whether the same sperm subpopulation was driving these correlations, we designed a double-labelling experiment to re-examine sperm samples previously found to harbour substantial DNA nicks (Fig. 5C). For this experiment, single-stranded DNA nicks were labelled via Nick translation with FITC, while accessible chromatin regions were labelled with ATTO565-dATP (a red fluorescent dye). The presence of 5-methylcytosine containing dNTPs in the initial Nick translation reaction ensured that DNA synthesized downstream of endogenous nicks was not further cleaved by Nt.CviPII during the subsequent reaction and thus should not lead to removal signal in these regions (Chin *et al.*, 2020). To control for possible incomplete repair following the initial labelling reaction, we also performed a sequential Nick translation reaction, where the incorporation of ATTO565-dATP is expected to be minimal. In these control experiments, we observed that sperm with very high levels of DNA nicking (labelled by FITC) did tend to show some increased ATTO565 signal (Fig. 5D, red points), suggesting that these represent sperm with extensively damaged DNA that T4 DNA ligase was not able to fully repair. In our double-labelling experiments, however, we observed multiple populations of sperm (Fig. 5D, blue points). In both total and swim-up sperm samples, we found sperm possessing both high DNA accessibility and high DNA nicking signal, as well as sperm with high DNA accessibility but limited DNA nicking (Fig. 5D), showing that a high level of DNA accessibility is not necessarily associated with increased DNA damage. We failed to detect a population of sperm with low DNA accessibility and high DNA nicking, but this may be caused by technical challenges as Nick translation may require at least some accessibility to enable sufficient incorporation of fluorescent label for detection.

## Discussion

Mammalian sperm chromatin is highly specialized for the effective and safe delivery of its genetic material to the oocyte, requiring packaging of the DNA into an especially densely compacted structure. Abnormalities in the condensation of sperm chromatin have been implicated in causing DNA fragmentation (for review, see Muratori and De Geyter, 2019), in fertilization failure (Sakkas *et al.*, 1996) and in the impaired formation of blastocysts during early embryogenesis (Fournier *et al.*, 2018). Using the novel NicE-view technology, we quantified for the first time levels of accessible DNA in human sperm samples from normozoospermic infertile individuals who had been able to generate few to many embryos after normal fertilization *in vitro*. The involvement of a maternal component was minimized by selecting female partners with normal ovarian reserve (normal antral follicle counts and AMH >10 pmol/l) and by the availability of at least five oocytes for fertilization. The current findings are the first to demonstrate that differences in sperm chromatin packaging, as given by differences in accessibility of sperm DNA, may impact the likelihood of pre-implantation embryonic development.

Our approach differs from most existing sperm assays in several ways. Firstly, the molecular underpinnings of the NicE-view assay are clearly understood. The nicking endonuclease used in this assay introduces a single-stranded DNA nick in accessible regions of the genome and surrounded open regions are then labelled by incorporation of fluorescent nucleotides. In contrast, CMA3 and AAB, two commonly used assays used to measure chromatin density, recognize and bind to features indirectly associated with DNA accessibility (Bianchi *et al.*, 1993; de Lamirande *et al.*, 2012).

Secondly, rather than scoring a few hundred sperm directly at the microscope (as recommended in the WHO manual; World Health Organization, 2021), we collected images of more than 3 million individual spermatozoa. We then used computational pipelines to identify the heads of each individual spermatozoon and to quantify the levels of accessible DNA present in each of these cells. Using this approach, we obtained high-confidence distributions of the levels of DNA accessibility within individual sperm samples. We find that the level of DNA packaging in single sperm varies dramatically within and between samples from individuals with differing reproductive outcomes (Fig. 1C).

Our results raise several interesting questions related to the generation of these varying chromatin states and their potential functional differences.

Firstly, the finding that sperm selected for motility via swim-up show a general increase in DNA accessibility was unexpected. Since its first description more than 50 years ago (Lopata *et al.*, 1976), the swim-up method is widely used as a method for isolation of motile sperm. The recovery of motile spermatozoa seems physiological, as it correlates with the migration of sperm through cervical mucus and with the fertilization rate in IVF (De Geyter *et al.*, 1988). Swim-up is also an effective approach to eliminate sperm with DNA fragmentation (Younglai *et al.*, 2001; Oguz *et al.*, 2018), which we confirmed in the Nick translation assay (Fig. 1C). The increase in highly accessible sperm following swim-up preparation may arise from two possible sources. The process of preparing sperm may alter chromatin, such that accessibility is increased, or alternatively, sperm with more accessible DNA may be enriched within the motile fraction of total sperm. Previous work has suggested that capacitation could increase the accessibility of sperm DNA (de Lamirande *et al.*, 2012). Comparison of NicE-view to capacitation in total and swim-up samples showed, however, no clear correlation (data not shown), suggesting that capacitation may not be the driver of swim-up associated increase in sperm chromatin accessibility.

Our data suggest that motile sperm may inherently possess increased chromatin accessibility compared to immotile sperm. A reason for this having not been previously appreciated may relate to differing labelling methodologies. We observe a clear bimodal distribution of signal in sperm using NicE-view, whereas CMA3, an existing assay for chromatin accessibility, gives a more continuous distribution (Stenz *et al.*, 2022). Likewise, we observe substantially stronger labelling with NicE-view compared to ATAC-see (Fig. 4B). Why NicE-view provides higher signal compared to these other methods is currently unclear and should be the subject of future research.

A second open question relates to functional differences between NicE-view^high^ and NicE-view^low^ sperm. Overall, the frequency of NicE-view^high^ sperm in both total and swim-up samples across all groups correlates negatively with conventional semen analysis parameters (Fig. 1C). Interestingly, our participant cohort was selected such that these conventional parameters were within the 5th percentile of the WHO fertile reference range (Cooper *et al.*, 2010). This suggests that even within these fertile ranges further subdivision of values may contain information related to sperm quality. Sperm samples containing very high frequencies of NicE-view^high^ sperm most commonly occurred in the LBGR sub-group, but the number of individuals with this pattern was limited even among this category to 5 out of 20, suggesting that in the other 75% of participants other sperm-borne or oocyte-related factors are responsible for the observed embryonic growth deficiencies. Further studies of a wider pool of participants are necessary to determine how commonly a high frequency of NicE-view^high^ sperm occurs in fertile and infertile populations. If this state remains infrequent in individuals with sperm of high developmental competence, NicE-view may become a tool for the prediction/explanation of abnormal embryonic development in ART.

Subjecting sperm samples to the swim-up preparation revealed differences that were not seen in total samples in more than 30% of individuals, independent of participant category (Fig. 2B). Our analysis suggests that increased DNA nicking occurs, together with decreased sperm concentration, in these samples, though double labelling shows that only a fraction of the sperm with high DNA accessibility also possess DNA nicks (Fig. 5D). The origin of these nicks may provide a further explanation as to the establishment of the NicE-view^high^ state that we observe in some sperm (discussed below). It remains to be investigated whether other sperm preparation methods may result in the selection of fewer spermatozoa with high DNA accessibility compared to swim-up.

The chromatin composition of haploid round spermatids is similar to that found in somatic nuclei, enriched for histone proteins and being transcriptionally active (Lewis *et al.*, 2003). During spermiogenesis, the chromatin state of haploid male germ cells undergoes dramatic re-configuration, with most histone proteins being removed and replaced by protamines (Lewis *et al.*, 2003), and transcription being globally silenced (Kierszenbaum and Tres, 1975). Topoisomerase enzymes, which maintain DNA topology, are important for this remodelling process (Laberge and Boissonneault, 2005). The enzymatic activity of Class I topoisomerases generates transient single-stranded DNA breaks (Krogh and Shuman, 2000), while Class II enzymes generate double-stranded breaks to relieve DNA supercoiling (Corbett and Berger, 2004). Enzymatic activity of both classes of topoisomerases is detected in mature human sperm (Har-Vardi *et al.*, 2007). These DNA breaks are usually rapidly repaired following topoisomerase reactions, but failure in this process could lead to persistent DNA damage (such as the Nick translation signal observed in some of our samples), and this damage could lead to impaired embryonic development (Nguyen *et al.*, 2023). Sperm in which the proper chromatin re-configuration process is aberrant may fail to fully incorporate protamines into their genomes, in addition to maintaining persistent DNA damage.

Whether the variation in chromatin state observed in mature sperm arises in the testis during spermatid development or arises later during epididymal transit or in the vas deferens or after ejaculation remains unknown. The absence of a correlation between abstinence time and NicE-view^high^ frequency (Fig. 2C) suggests that this state does not increase with sperm ageing through prolonged abstinence. Characterization of the chromatin of spermatids or testicular sperm by NicE-view would be interesting to address this question. Testicular sperm functions more effectively in ART in individuals where DNA fragmentation in ejaculated sperm is elevated (Esteves *et al.*, 2015; Bradley *et al.*, 2016). Our cohort does not possess globally increased levels of sperm DNA fragmentation as measured by TUNEL (Table 1), but if NicE-view^high^ sperm are not found in testicular sperm, it is intriguing to wonder if isolation of sperm via testicular sperm extraction could be used in cases where pre-implantation development following ART fails.

An open question related to this work is whether specific genomic regions are labelled by NicE-view and whether these regions differ between individuals. To identify such regions, it would be possible to use biotin (in place of fluorescence) to label accessible DNA and sequence such labelled regions (NicE-seq; Ponnaluri *et al.*, 2017; Chin *et al.*, 2020, 2023). A challenge to such an approach is that these measurements generate an averaged profile of many cells. Hence, NicE-view^low^ and NicE-view^high^ sperm would be mixed unless a purification approach for such samples could be performed. Future work is needed to solve these challenges.

## Supplementary Material

deaf081_Supplementary_Data_File

deaf081_Supplementary_Figure_S1

deaf081_Supplementary_Figure_S2

deaf081_Supplementary_Figure_S3

deaf081_Supplementary_Figure_S4

deaf081_Supplementary_Figure_S5

## Data Availability

Quantitation of imaging data and code used for analysis are available at Zenodo (10.5281/zenodo.12526509).
