# Supplementary material for: Normozoospermic infertile men possess subpopulations of sperm varying in DNA accessibility, relating to differing reproductive outcomes
Source: Hum Reprod. 2025 May 16;40(7):1266–81. doi: 10.1093/humrep/deaf081 (PMC12222617; doi:10.1093/humrep/deaf081)
Supplement: deaf081_Supplementary_Data_File [file deaf081_supplementary_data_file.pdf]

OXFORD
human reproduction

Human Reproduction, 2025, 1–2
https://doi.org/10.1093/humrep/deaf081
Supplementary Data

Supplementary Data File S1

Semen analysis methodology checklist for authors, reviewers, and editors (modified from Björndahl *et al.*, 2022)

This checklist was published in *Human Reproduction* (Björndahl *et al.* 2022—https://doi.org/10.1093/humrep/deac189).

This checklist is based on the ISO Standard on basic semen examination and the current WHO recommendations (WHO6) [1,2], and on general scientific standards. Full compliance requires that all boxes are ticked.

A deviation from this checklist does not necessarily mean that the study cannot be published, but all deviations must be declared in the Materials and methods section of the manuscript, including their impact on accuracy and measurement uncertainty of the data, in order to allow the reader to evaluate the quality of the analyses performed. For studies not reporting all characteristics of a basic semen examination, the checklist includes the option 'Not applicable to the study'.

Investigations that would be subject to the requirements in this checklist can roughly be classified as clinical (evaluating patient treatment, diagnostic classification, or predictive powers of certain assessments), experimental (e.g. exposure of spermatozoa to different compounds or *in vitro* treatments) [3,4], or epidemiological (evaluating variations in semen characteristics or effects of exposure populations to certain compounds or other circumstances).

Any scientific rationale for not complying with the guidelines, which is not included in the Materials and methods section of the manuscript, must be substantiated to the Editor and Reviewers.

References

1. World Health Organization. *WHO Laboratory Manual for the Examination and Processing of Human Semen*, 6th edn. Geneva: World Health Organization, 2021.
2. International Standards Organization. *ISO 23162:2021 Basic Semen Examination—Specification and Test Methods*. Geneva: International Standards Organization, 2021.
3. Mortimer D, Barratt CLR, Bjöorndahl L, de Jager C, Jequier AM, Muller CH. What should it take to describe a substance or product as 'sperm-safe'. *Hum Reprod Update* 2013;**19(Suppl 1)**:i1–i45.
4. Sanchez-Pozo MC, Mendiola J, Serrano M, Mozas J, Bjorndahl L, Menkveld R, Lewis SEM, Mortimer D, Jorgensen N, Barratt CLR *et al.*; Special Interest Group in Andrology (SIGA) of the European Society of Human Reproduction and Embriology. Proposal of guidelines for the appraisal of SEMen QUAlity studies (SEMQUA). *Hum Reprod* 2013;**28**:10–21.

1. Patients ☐ Not applicable to the study

■ 1.1 The studied population (e.g. patients or volunteers) has been declared in the manuscript, together with the recruitment method and inclusion and exclusion criteria. In a study concerning couples being investigated for infertility, the following is specified in the manuscript: fertility status of female partner; and primary, secondary, or other level of investigation of the man.

■ 1.2 If used in the manuscript, the term 'male factor' is completely defined.

■ 1.3 Reference limits provided in WHO5 or 5th percentile of distribution of semen examination results in WHO6 have not been used to characterize subjects as infertile, subfertile, or fully fertile.

2. General aspects ☐ Not applicable to the study

■ 2.1 Patients were instructed to maintain 2–7 days of sexual abstinence before collecting an ejaculate for investigation.

■ 2.2 Patients were informed about the importance of reporting any missed early ejaculate fractions, and their responses were noted on the laboratory record.

■ 2.3 For specimens not collected at the laboratory, patients were instructed to avoid cooling (under 20°C) or heating (above 37°C) of the semen specimen during transport to the laboratory.

■ 2.4 In the laboratory, specimens were kept at 37°C before initiation of and during the analysis in case of sperm motility assessment.

■ 2.5 For specimens collected adjacent to the laboratory, analysis was initiated after completion of liquefaction and within 30 min after ejaculation. If some of the specimens were collected at the laboratory and others collected at home the influence on the data is declared and discussed in the manuscript.

■ 2.6 Liquefaction was first checked within 30 min after ejaculation.

■ 2.7 Volume was determined either by weighing or using a wide-bore volumetric pipette.

■ 2.8 Viscosity was measured using either a wide-bore pipette or a glass rod.

■ 2.9 All staff members who performed the analyses have been trained in basic semen analysis (ESHRE Basic Semen Examination Course—or equivalent—with further in-house training to establish competency), and participate regularly in internal quality control.

■ 2.10 When more than one method is recommended for a particular characteristic (e.g. to measure volume), only one was used in the study. For a multicentre study, all laboratories used the same method or variable methods are declared in the manuscript.

3. Sperm concentration assessment ☐ Not applicable to the study

☐ 3.1 Semen aliquot to be diluted for sperm concentration assessment was taken with a positive displacement pipette (i.e. a 'PCR pipette') using a recommended diluent (state which diluent: ____________________).

■ 3.2 Only standard dilutions were used (1:50, 1:20 or 1:10, i.e. 1 + 49, 1 + 19 or 1 + 9).

☐ 3.3 Sperm concentration was assessed using haemocytometers with improved Neubauer ruling.

□ 3.4 Haemocytometers were allowed to rest for 10–15 min in a humid chamber to allow sedimentation of the suspended spermatozoa onto the counting grid before counting.

■ 3.5 Sperm counting was done using phase contrast microscope optics (200–400×).

■ 3.6 Comparisons were made between duplicate counts, and counts were re-done when the difference exceeded the acceptance limits.

■ 3.7 Typically at least 200 spermatozoa were counted in each of the duplicate assessments.

4. Sperm motility assessment □ Not applicable to the study

■ 4.1 Motility assessments were performed at 37°C ± 0.5°C.

■ 4.2 Motility assessments were initiated within 30–60 min after sample collection

■ 4.3 Motility assessments were performed using phase contrast microscope optics (200–400×).

■ 4.4 Sperm motility was classified using a four-category scheme: rapid progressive, slow progressive, non-progressive, and immotile.

■ 4.5 Motility assessments were done in duplicate and compared; counts were re-done on new preparations when the difference between duplicates exceeded the acceptance limits.

□ 4.6 The wet preparation was made using a drop of ___ μl and a ___ × ___ mm coverslip to give a depth of ____ μm (must be at least 10 μm depth, but not too deep so as to allow spermatozoa to move freely in and out of focus; typically ~20 μm).

■ 4.7 At least 200 spermatozoa were assessed in each duplicate motility count.

■ 4.8 At least five microscope fields of view were examined in each duplicate count.

5. Sperm vitality assessment □ Not applicable to the study

■ 5.1 A validated supravital stain, appropriate to the type of microscope optics employed, was used to assess sperm vitality.

■ 5.2 At least 200 spermatozoa were evaluated.

■ 5.3 Assessments were done under high magnification (×1000–1250) using a 100× high-resolution oil immersion objective and bright field microscope optics (Köhler illumination).

6. Sperm morphology assessment □ Not applicable to the study

■ 6.1 Tygerberg Strict Criteria were used for the evaluation of human sperm morphology. (Another classification could be used for scientific studies with specific aims if the classification is described or referenced. Depending on the aim of the study, the evaluation of particular abnormal forms might be useful.)

■ 6.2 Abnormalities are recorded for the four defined regions of the spermatozoon (head, neck/midpiece, tail, and cytoplasmic residue).

□ 6.3 The Papanicolaou staining method adapted for the assessment of human sperm morphology was used. For specific aims, other staining methods could be used but must then be declared and explained.

■ 6.4 At least 200 spermatozoa were assessed in each ejaculate.

■ 6.5 Assessments were done under high magnification (×1000–1250) using a 100× high-resolution oil immersion objective and bright field microscope optics (Köhler illumination).

7. External Quality Assessment (EQA)

■ 7.1 The laboratory participated in EQA for the semen examination methods used to obtain data for the study.

■ 7.2 Name of the EQA scheme: QuADeGA_________________

8. Other findings □ Not applicable to the study

■ 8.1 The presence of abnormal clumping (aggregates and agglutinates) was recorded.

■ 8.2 Abnormal viscosity was recorded.

9. Analysing data □ Not applicable to the study

■ 9.1 The actual duration of sexual abstinence (in ‘hours’ or ‘days’) was recorded for each specimen and included in the data reported in the manuscript.

■ 9.2 As a minimum in clinical studies, semen volume, sperm concentration, total number of spermatozoa per ejaculate, and abstinence time are given to reflect sperm production and output; only samples identified as having been collected completely were included in the study.

■ 9.3 Confounding factors have been considered for statistical analysis: e.g. abstinence time and age, to consider secular or geographical variations in sperm concentration or sperm count.

■ 9.4 If appropriate, optional biochemical markers for prostatic, seminal vesicular, and epididymal secretions were analysed and reported, both as concentration and total amount.

■ 9.5 Signs of active infection/inflammation were noted and considered in the analysis of data in the study (e.g. presence of non-germ line round cells, inflammatory cells, impaired sperm motility, possibly also anti-sperm antibodies or reduction of secretory contributions).

10. Data Repository

□ 10.1 For the sake of transparency, all data without identification of individual patients or research participants have been saved to a trusted online repository, and there is a statement of this in the Results section of the manuscript.

Declaration by the corresponding author:
The information provided in this checklist is solemnly declared to be true.
Signature:______________________Date:______________________
Name:_________________________
Affiliation:____
