# Supplementary material for: Normozoospermic infertile men possess subpopulations of sperm varying in DNA accessibility, relating to differing reproductive outcomes
Source: Hum Reprod. 2025 May 16;40(7):1266–81. doi: 10.1093/humrep/deaf081 (PMC12222617; doi:10.1093/humrep/deaf081)
Supplement: deaf081_Supplementary_Figure_S1 [file deaf081_supplementary_figure_s1.pdf]

OXFORD
human reproduction
Human Reproduction, 2025, 1–1
https://doi.org/10.1093/humrep/deaf081
Supplementary Data

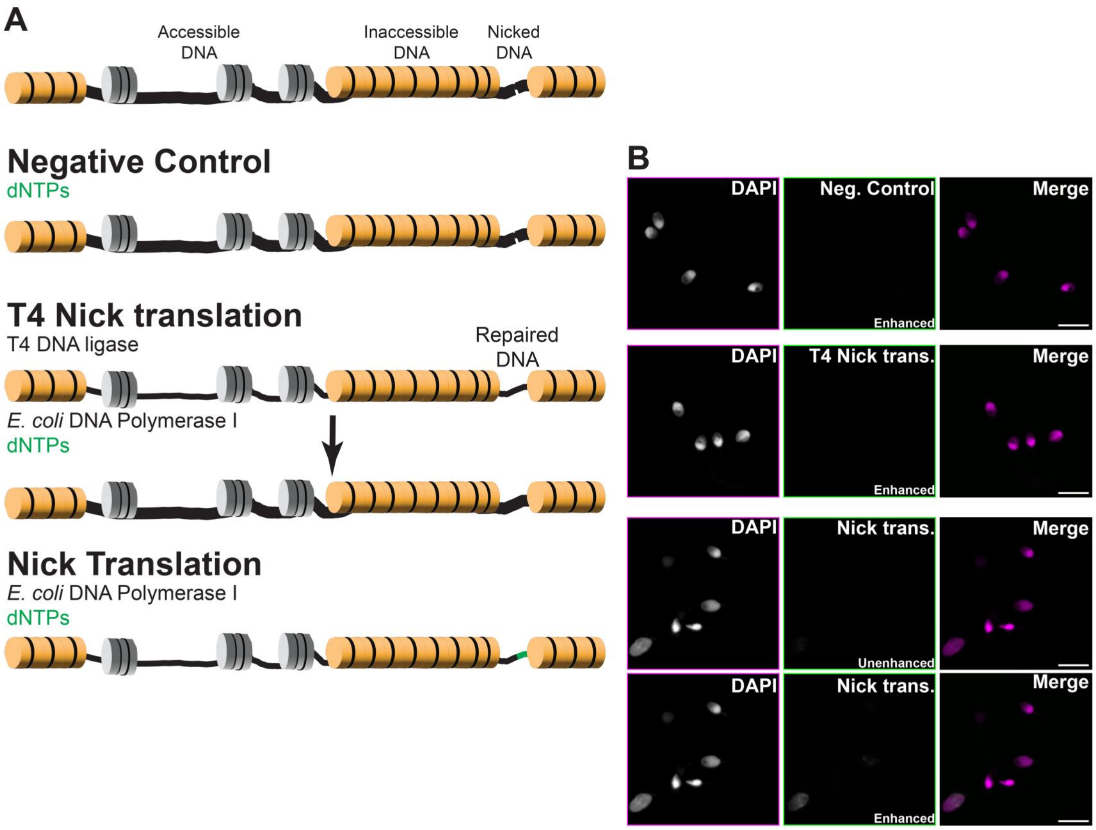

Supplementary Figure S1 (Related to Fig. 1). Control staining shows low background of NicE-view and effectiveness of T4 DNA ligase. (**A, B**) as in Fig. 1A and B but including two additional staining conditions: Negative control (incubated with fluorescent dNTPs but no enzymes) and T4 Nick translation (treated with T4 DNA ligase prior to Nick translation reaction). For Nick translation samples, unenhanced refers to images with no adjustment to brightness and contrast, while enhanced refers to images where brightness and contrast have been enhanced to make the Nick translation signal more visible. Scale bars = 10 μm.
