# Supplementary figures and images for: Normozoospermic infertile men possess subpopulations of sperm varying in DNA accessibility, relating to differing reproductive outcomes
Source: Hum Reprod. 2025 May 16;40(7):1266–81. doi: 10.1093/humrep/deaf081 (PMC12222617; doi:10.1093/humrep/deaf081)

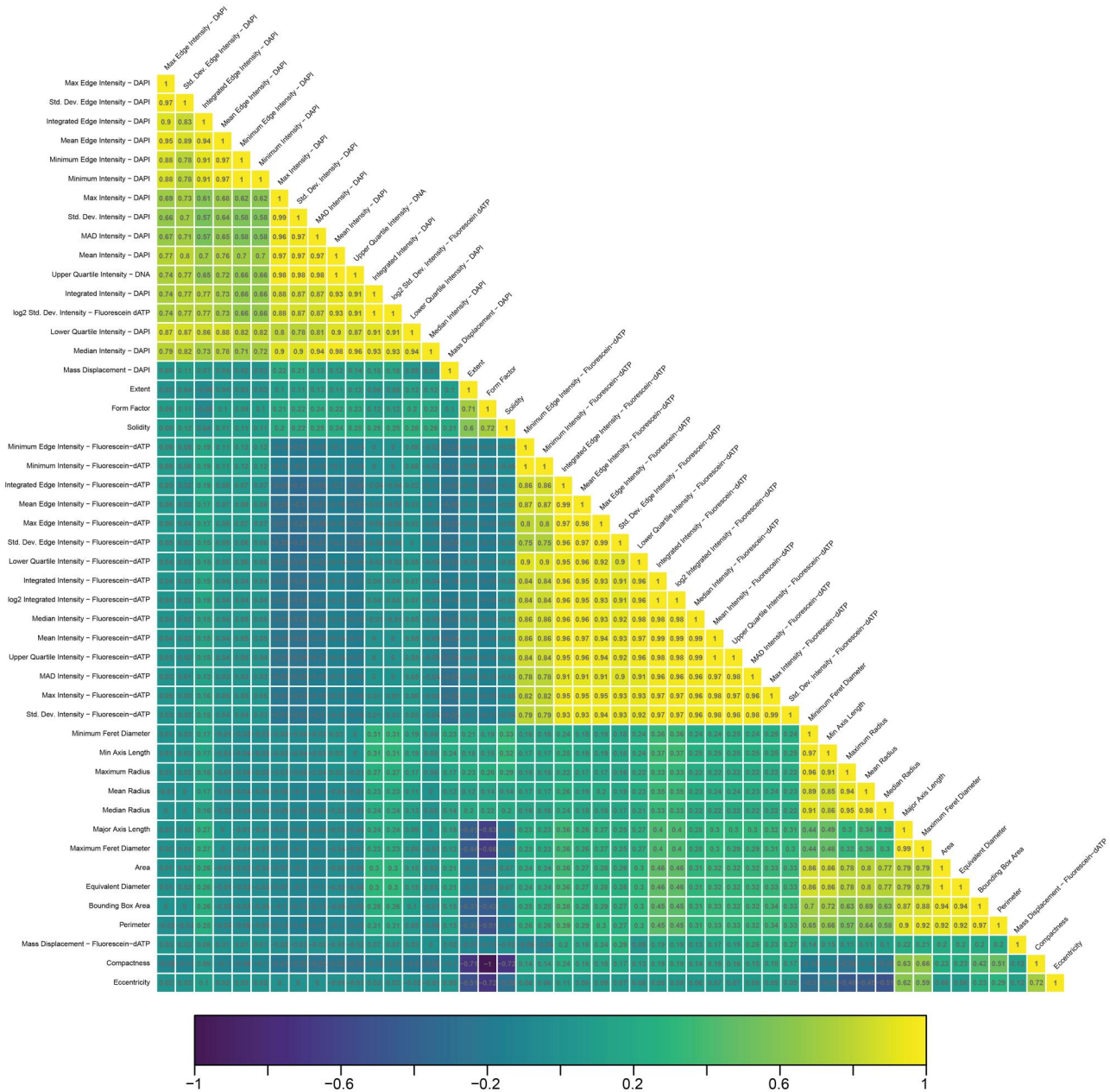

Supplement: deaf081_Supplementary_Figure_S2 [file deaf081_supplementary_figure_s2.pdf]
