# Supplementary material for: Normozoospermic infertile men possess subpopulations of sperm varying in DNA accessibility, relating to differing reproductive outcomes
Source: Hum Reprod. 2025 May 16;40(7):1266–81. doi: 10.1093/humrep/deaf081 (PMC12222617; doi:10.1093/humrep/deaf081)
Supplement: deaf081_Supplementary_Figure_S3 [file deaf081_supplementary_figure_s3.pdf]

OXFORD
human reproduction

Human Reproduction, 2025, 1–1
https://doi.org/10.1093/humrep/deaf081
Supplementary Data

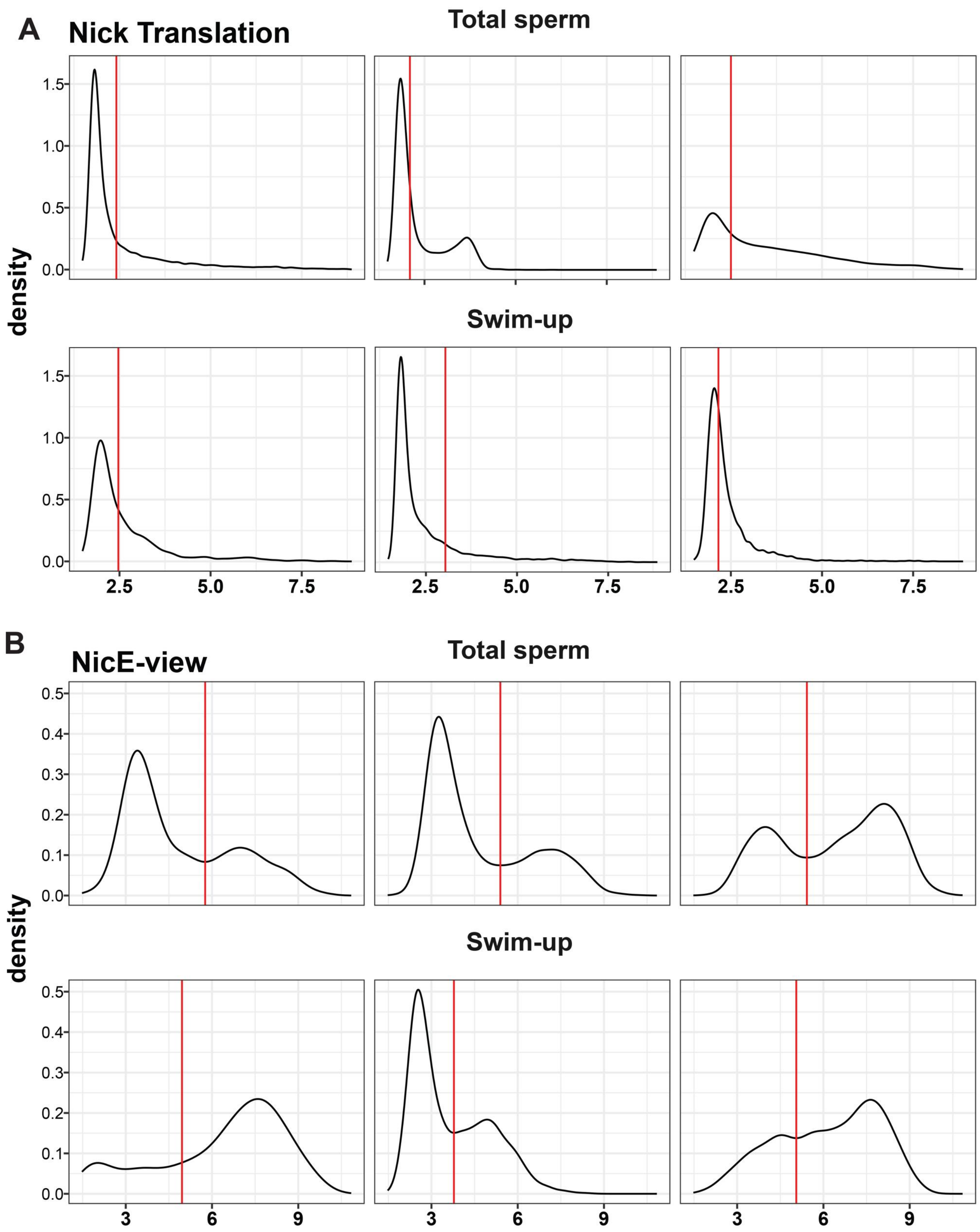

Supplementary Figure S3 (Related to Fig. 2). Schematic diagrams illustrating the setting of quantification thresholds for Nick translation, NicE-view, and T4 NicE-view signals. (**A**) Density plots showing threshold values (red lines) for Nick translation staining in three individuals. (**B**) Density plots showing threshold values (red lines) for NicE-view staining in three individuals (same as in (A)). Upper plots show values for Total sperm, while lower panels show values for Swim-up sperm from same individuals.
