# Supplementary material for: Normozoospermic infertile men possess subpopulations of sperm varying in DNA accessibility, relating to differing reproductive outcomes
Source: Hum Reprod. 2025 May 16;40(7):1266–81. doi: 10.1093/humrep/deaf081 (PMC12222617; doi:10.1093/humrep/deaf081)
Supplement: deaf081_Supplementary_Figure_S4 [file deaf081_supplementary_figure_s4.pdf]

Human Reproduction, 2025, 1–1
https://doi.org/10.1093/humrep/deaf081
Supplementary Data

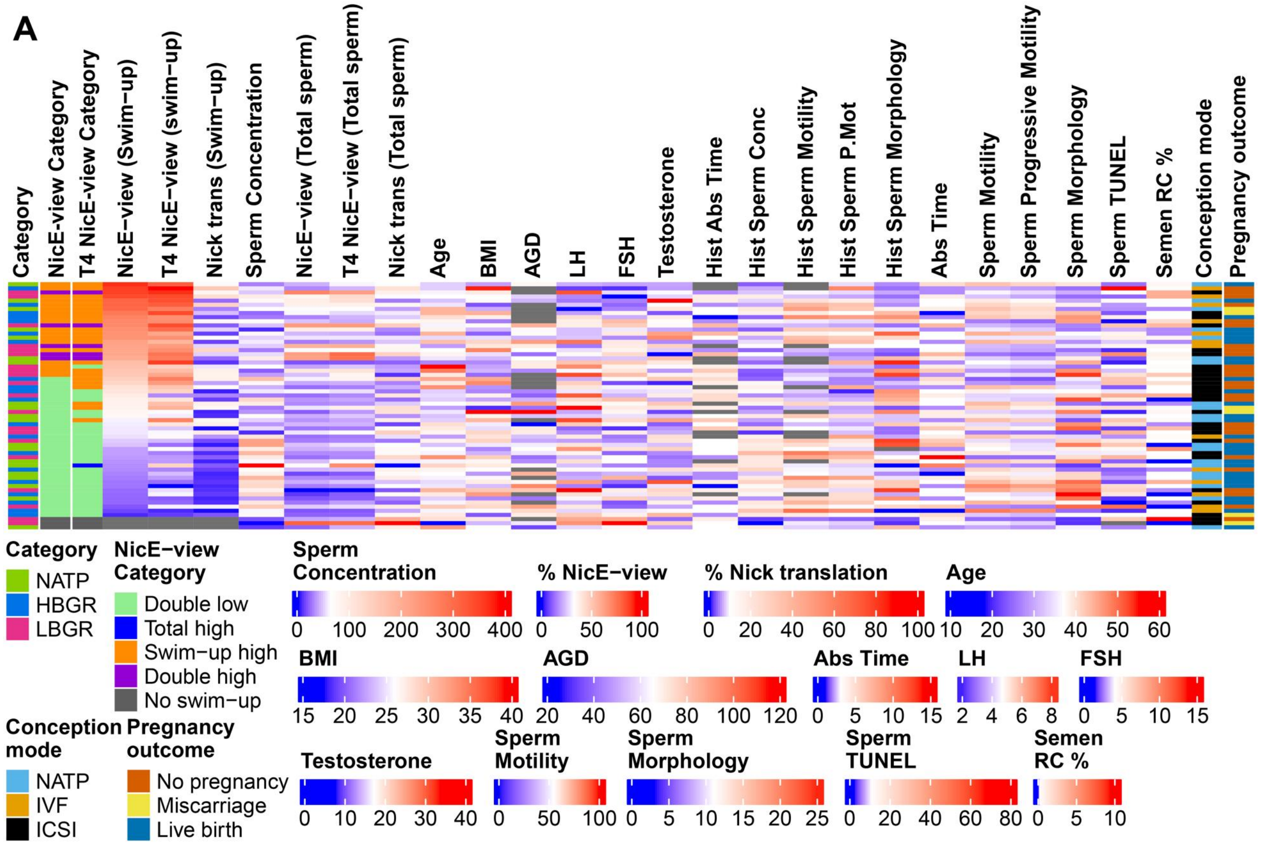

Supplementary Figure S4 (Related to Fig. 3). No correlation of NicE-view frequency with demographic or hormonal parameters in participant cohort. Heatmap as in Fig. 3C showing additional parameters related to participant cohort. Data are ordered by % NicE-view^{high} sperm in swim-up sample.
