# Supplementary material for: Normozoospermic infertile men possess subpopulations of sperm varying in DNA accessibility, relating to differing reproductive outcomes
Source: Hum Reprod. 2025 May 16;40(7):1266–81. doi: 10.1093/humrep/deaf081 (PMC12222617; doi:10.1093/humrep/deaf081)
Supplement: deaf081_Supplementary_Figure_S5 [file deaf081_supplementary_figure_s5.pdf]

OXFORD
human reproduction

Human Reproduction, 2025, 1–1
https://doi.org/10.1093/humrep/deaf081
Supplementary Data

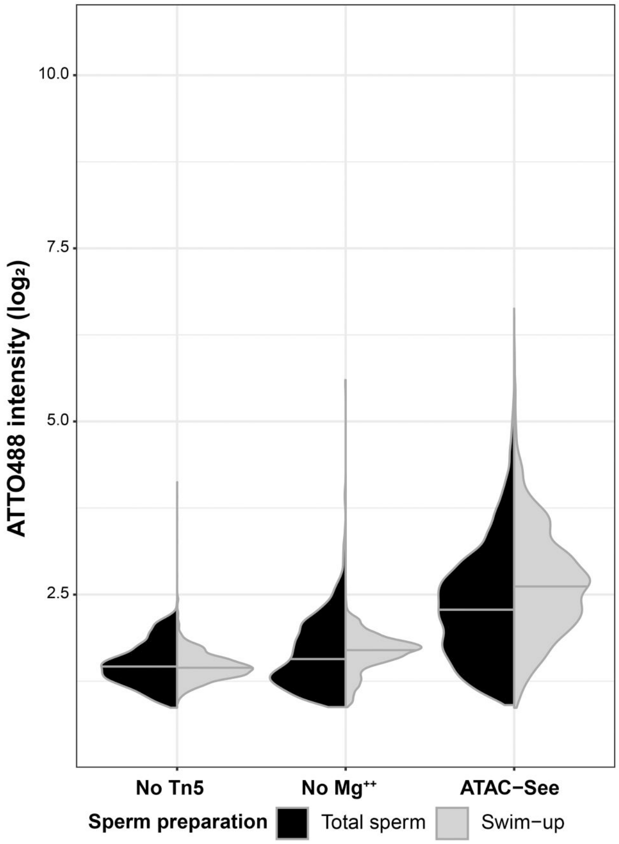

Supplementary Figure S5 (Related to Fig. 4). 0Tn5 enzymatically integrates labelled oligonucleotides into sperm chromatin. (**A**) Split violin plot showing control reactions for ATAC-see. No Tn5 refers to incubation with labelled oligonucleotides but no enzyme; No Mg++ refers to incubation with labelled oligonucleotides and Tn5 transposase without Magnesium (a necessary Tn5 cofactor). Data are pooled from five individuals.

© The Author(s) 2025. Published by Oxford University Press on behalf of European Society of Human Reproduction and Embryology.
This is an Open Access article distributed under the terms of the Creative Commons Attribution License (https://creativecommons.org/licenses/by/4.0/), which permits unrestricted reuse, distribution, and reproduction in any medium, provided the original work is properly cited.
Human Reproduction, 2025, 00, 1–23
https://doi.org/10.1093/humrep/deaf081
Original Article
